# Essential Role of the A’α/Aβ Gap in the N-Terminal Upstream of LOV2 for the Blue Light Signaling from LOV2 to Kinase in Arabidopsis Photototropin1, a Plant Blue Light Receptor

**DOI:** 10.1371/journal.pone.0124284

**Published:** 2015-04-17

**Authors:** Sachiko Kashojiya, Koji Okajima, Takashi Shimada, Satoru Tokutomi

**Affiliations:** 1 Department of Biological Science, Osaka Prefecture University, Sakai, Osaka, Japan; 2 Life Science Research Center, SHIMADZU Corporation, Tokyo, Japan; USDA-ARS, UNITED STATES

## Abstract

Phototropin (phot) is a blue light (BL) receptor in plants and is involved in phototropism, chloroplast movement, stomata opening, etc. A phot molecule has two photo-receptive domains named LOV (Light-Oxygen-Voltage) 1 and 2 in its N-terminal region and a serine/threonine kinase (STK) in its C-terminal region. STK activity is regulated mainly by LOV2, which has a cyclic photoreaction, including the transient formation of a flavin mononucleotide (FMN)-cysteinyl adduct (S390). One of the key events for the propagation of the BL signal from LOV2 to STK is conformational changes in a Jα-helix residing downstream of the LOV2 C-terminus. In contrast, we focused on the role of the A’α-helix, which is located upstream of the LOV2 N-terminus and interacts with the Jα-helix. Using LOV2-STK polypeptides from *Arabidopsis thaliana* phot1, we found that truncation of the A’α-helix and amino acid substitutions at Glu474 and Lys475 in the gap between the A’α and the Aβ strand of LOV2 (A’α/Aβ gap) to Ala impaired the BL-induced activation of the STK, although they did not affect S390 formation. Trypsin digested the LOV2-STK at Lys603 and Lys475 in a light-dependent manner indicating BL-induced structural changes in both the Jα-helix and the gap. The digestion at Lys603 is faster than at Lys475. These BL-induced structural changes were observed with the Glu474Ala and the Lys475Ala substitutes, indicating that the BL signal reached the Jα-helix as well as the A’α/Aβ gap but could not activate STK. The amino acid residues, Glu474 and Lys475, in the gap are conserved among the phots of higher plants and may act as a joint to connect the structural changes in the Jα-helix with the activation of STK.

## Introduction

Plants use light as a signal to conduct many physiological responses as well as a source of energy. Phototropin (phot) [1] is one of the major blue light receptors in plants [2] and regulates phototropism [3], chloroplast movement [4–6], stomata opening [7] and so on to optimize the photosynthetic efficiency of plants. Most plants have two isoforms of phot named phot1 and phot2 [8]. In *Arabidopsis thaliana* (*At*), phot1 mediates physiological responses, such as phototropism, over a broad range of light intensity, whereas phot2 acts as a high light sensor [4]. Phot consists of approximately 1000 amino acid residues, has two LOV (LOV1 and LOV2) domains in its N-terminal region, a serine/threonine kinase (STK) in its C-terminal region [3] and works as a BL-regulated protein kinase. LOV forms a subfamily of the Per-Arnt-Sim (PAS) super family [9]. The LOV of phots binds an oxidized flavin mononucleotide (FMN) non-covalently in a pocket formed by α-helices and a 5-stranded β-sheet scaffold characteristic with the PAS fold [10] and works as a light perceptive domain through a cyclic photoreaction of the FMN with a conserved neighbor cysteine residue [11]. The C-terminal STK is classified as subfamily VIII of the protein kinase AGC group, and the amino acid residues required for the kinase activities are highly conserved [12]. Phot undergoes autophosphorylation in a light dependent manner in which LOV2 is a main regulator of the STK activity [3,13,14]. Recently, ABCB19 [15], PKS4 [16] and BLUS1 [17] have been found as the substrates of the phot kinase involved in the BL signaling in Arabidopsis.

Molecular events initiated by BL in the LOV2 of phot have been well studied by many biophysical techniques using LOV2-containing small polypeptides. Upon BL irradiation, a transient covalent bond is formed between the FMN and the Cys residue conserved near the FMN (S390 intermediate) after intersystem crossing to the triplet-excited state [18]. S390 reverts to the ground state (D450) thermally with time constants from seconds to minutes [19]. The decay time of S390 is correlated with the duration of the BL-induced activation of STK [20] and may reflect the diverse photosensitivity of phots working under different light conditions [20,21]. The adduct formation, however, induces only small conformational changes in the LOV, which was observed by X-ray crystallography, [22,23], small angle X-ray scattering (SAXS) [24] and transient grating (TG) [25]. The X-ray crystallography studies on the LOV2 of *Adiantum* neochrome1 revealed BL-induced flipping of the Gln1029 residue interacting with N5 of the FMN isoalloxazine ring [22,23]. Substitution of this Gln to Leu resulted in the loss of conformational changes as detected by Fourier transform infrared (FTIR) spectroscopy [26]. Thus, the Gln corresponding to Gln575 in *At* phot1 and Gln 513 in *Avena sativa* (*As*) phot1 [27] is one of the key residues responsible for the structural changes in LOV2.

In contrast, a large conformational change has been reported to occur in an α-helix named J that resides downstream of the C-terminus of the LOV2 and runs on the surface of the β-sheet [28]. NMR studies on the *As* phot1 LOV2-Jα polypeptide of revealed that BL induced a slight structural change and successive dissociation from the LOV2 and unfolding of the Jα-helix [29–31]. In *As* phot1, Ile532, Ala536, Ile539 and Asp540 in the Jα-helix contribute to keeping the conformation of Jα-helix intact. Comparison of the crystal structures of *As* phot1 LOV2-Jα prepared under dark and light conditions suggested that the BL signal perceived by FMN propagates to the middle part of the Jα-helix through rearrangement of the hydrogen bond network between the β-sheet and the Jα-helix [28]. *In vitro* autophosphorylation assay of *At* phot1 prepared from insect cells showed that the substitution at Ile608 in the Jα-helix, corresponding to Ile539 in *As* phot1, to Glu impaired the light regulation of STK activity [30]. Structural change in the Jα-helix is, therefore, thought to be a key process for the intramolecular signal transduction from LOV2 to STK.

**Fig 1:**
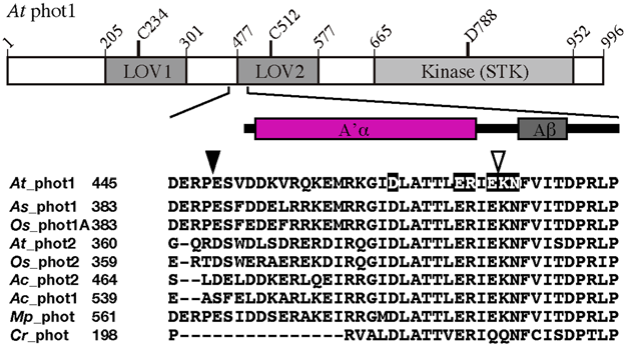
Schematic drawing of *At* phot1 (upper panel) and the secondary structure around A’α-helix region. Alignment of the amino acid sequences of phot in the A’α-helix regions using ClustalX. *Arabidopsis thaliana*, *At*; *Avena sativa*, *As*; *Oryza sativa*, *Os*; *Adiantum capillus-veneris*, *Ac*; *Marchantia*, *polymorpha*, *Mp*; *Chlamydomonas reinhardtii*, *Cr*. Filled and open arrow heads indicate the N-ends of LOV2-STK and ΔA’α constructs, respectively. Amino acid residues substituted in this study are highlighted.

In addition to the Jα-helix, recent studies have identified the involvement of another α-helix named A’ in intramolecular signaling. A’α-helix is located upstream of the N-terminus of LOV2. In green algae *Chlamydomonas reinhardtii* (*Cr*) phot, amino acid mutations in this helix affected the regulation of STK activity [32]. The amino acid sequences for the A’α-helix are conserved among higher plant phots (Fig 1) suggesting that the helix may function in the intramolecular signal transduction from LOV2 to STK. In fact, an amino acid mutation in the A’α-helix region impaired *in vivo* phot1 signaling in the tomato [33]. The *As* phot1 LOV2-Jα polypeptide used in the previous crystal structure determination contained 7 amino acid residues in the A’α-helix region that forms a short 4 amino acid helix [28]. Based on this structure, molecular dynamics (MD) calculations proposed that the A’α-helix plays a role in intramolecular light signaling with the Jα-helix [34,35]. Recently, a crystal structure was determined with *At* phot1 LOV2-Jα with a larger number, 21, of amino acid residues in the A’α-helix region [36]. In contrast to the previous monomeric *As* phot1 LOV2-Jα with the short A’α-helix, *At* phot1 LOV2-Jα forms a dimer and each subunit has a longer A’α-helix. The N-terminal extension serves as the dimer interface by configuring a short α-helical coiled coil with a scissor-like shape. The Jα-helix attaches on the surface of the β-sheet of the LOV2 in a similar fashion as the previous *As* phot1 LOV2-Jα. Both helices in a subunit orient in a similar direction and interact with each other at their edges. All of the LOV2-containing polypeptides of *At* phot1 used so far include the longer A’α-helix. A TG study on the *At* phot1 LOV2-Jα polypeptide showed that the conformational change in the Jα-helix has a faster reaction rate than that of the A’α-helix [37]. The signaling process from FMN to A’α-helix and its communication with the signaling to the Jα-helix is obscure. Furthermore, the involvement of these processes in the signaling from LOV2 to STK is unknown. Thus, the function of the A’α-helix in these intramolecular BL signaling processes is to be examined in detail.

To uncover the molecular processes involved in the BL signal transduction from the chromophore to not only the Jα-helix but also to the STK, an appropriate assay system is requisite. Recently, we have developed a useful assay system for that purpose. The system is composed of a set of a BL-regulated kinases and an artificial substrate derived from parts of *At* phots. The LOV2-kinase of phot1 covers the amino acids from the A’α-helix to the C-terminal end, including LOV2, linker and kinase domain. They showed a typical photocycle of LOV2 upon BL excitation and a kinase activity on the substrate consisting of an N-terminal region of *At* phot1 in a light dependent manner [20,38]. To elucidate the role of the A’α-helix in the BL signaling described above, we studied effects of the deletion of the A’α-helix and the amino acid substitutions in this region on the photochemistry and the kinase regulation by BL using the LOV2-STK preparation of *At* phot1. Interestingly, we found that the gap between the A’α-helix and the Aβ-strand of LOV2 (A’α/Aβ gap) play a critical role as well as the A’α-helix.

## Materials and Methods

### Construction of expression vectors

WT, C512A and D788A were prepared as described previously [38]. For A’α truncated LOV2-STK (ΔA’α, 475–996), the truncation in the preparation included Glu474 at the A’α/Aβ gap, *Nde*I site was inserted into the pET28a_WT vector by PCR-based site-directed mutagenesis using primer sets (S1 Table). After *Nde*I treatment, vector was self-ligated. For the D465A, E471A, R472A, E474A, E474W, E474K, K475A and N476A amino acid substitutes, PCR-based site-directed mutagenesis was performed with the primer sets in S1 Table. The vectors for the LOV2 with Jα-helix (LOV2-Jα, 449–614) and the LOV2 with a full-length linker between LOV2 and STK domain (LOV2-linker, 449–661) were prepared as described previously [38].

### Expression and Purification of recombinant proteins

Vectors were transformed into *E*. *coli* strain BL21 (DE3). Cells were cultured in LB medium containing 30 μg mL^-1^ kanamycin for 6 h at 37°C. Overexpression was induced with 0.04 mM isopropyl β-D-thiogalactopyranoside for 24 h at 18°C. Cells were harvested by centrifugation and stored at -80°C until use. The cells were thawed and re-suspended into the extraction buffer (20 mM HEPES pH 7.5, 0.5 M NaCl 10% (w/v) glycerol) containing 2 mM phenylmethylsulphonyl fluoride and final 1 mg mL^-1^ Lysozyme, and then incubated for 30 min on ice. Cells were lysed by sonication and centrifuged (100,000 x g for 30 min at 4°C). The supernatant was loaded on to a nickel affinity column (Ni-Sepharose High Performance, GE), and protein was eluted with buffer contacting 500 mM imidazole after the resin was washed with buffer containing 30 mM imidazole. The partially purified protein was further purified with size-exclusion column chromatography (Superdex 200 pg, GE). ΔA’α and the other polypeptides were diluted into a buffer containing 500 and 100 mM NaCl, respectively [38]. The apparent molecular weights of WT and mutants estimated from elution profiles of the size-exclusion column chromatography were almost the same, 95 kDa, suggesting that they are in the same oligomeric form (S1 Fig). Their purities were estimated by the Coomassie Brilliant Blue (CBB)-staining of the SDS-PAGE gels. The purities were approximately 90% and more than 95% for ΔA’α and the other polypeptides, respectively.

### Phosphorylation Assay

Phosphorylation assay was performed in the reaction buffer (Tris-HCl pH 7.5, 1 mM EGTA, 10% (w/v) glycerol-containing 100 mM NaCl) containing 10 mM MgCl_2_, 10 μM ATP and 3.7 kBq of [γ-^32^P] ATP. In the case of ΔA’α, the concentration of NaCl was 500 mM. Sample was incubated with an artificial substrate, P1Nt (N-terminal 1–463 fragment of *At* phot1) [38] at 20°C for 30 min under BL or in the dark. The irradiation was performed with a blue LED illuminator (ISL-150X150-88, CCS Inc., Japan, λ_max_ at 475 nm, 34 μmol m^-2^ s^-1^). The reaction was stopped by adding a SDS-PAGE sample buffer and then boiling for 3 min. Samples were separated by SDS-PAGE and phosphorylated bands were visualized with imaging plates (Fujifilm, Japan) and STORM scanner (GE Healthcare, Japan) [38]. Phosphorylation assays were performed tree times and the reproducibility was confirmed.

### Spectroscopy

UV-Vis absorption spectra were recorded with a spectrophotometer (3310, Hitachi-hitec, Japan) equipped with a thermoelectric cell holder. The sample in a cell was irradiated with a handmade blue LED illuminator (LUXEON star, λ_max_ = 465 nm). BL-excited spectra were recorded immediately after the flash BL irradiation (~250 μmol m^-2^ s^-1^, for 2 s). Dark reversion kinetics was monitored at 447 nm at 20°C [38]. Half-decay times were averaged for three data.

### Proteolysis assay and amino acid sequencing

Trypsin (GE Healthcare, Japan) solution was added to a polypeptide solution of 0.4 mg mL^-1^ to give the final trypsin concentration of 10 μg mL^-1^. Digestion was performed at 20°C under BL irradiation or in the dark and was stopped by boiling the solutions for 3 min after adding a concentrated SDS-PAGE sample buffer. Digested polypeptides were separated by SDS-PAGE and stained with CBB. Proteolysis assays were performed two times and the reproducibility was confirmed.

The amino acid sequences of the polypeptides separated in SDS-PAGE gels were determined by mass spectrometry (MS). Polypeptides were excised and digested in-gel with trypsin (Promega, USA) after reductive alkylation in 100 mM ammonium bicarbonate solution containing 10 mM dithiothreitol and 55 mM iodoacetamide. After incubation at 37°C for 12 h in 50 mM ammonium bicarbonate and 1 mM calcium chloride, tryptic polypeptides were desalted with a ZipTip μC18 (Millipore, USA). The peptides were applied on the matrix-assisted laser desorption/ionization (MALDI) analysis using recrystallized 2,5-dihydroxybenzoic acid (Shimadzu GLC, Japan) in 50% acetonitrile at a concentration of 10 mg mL^-1^ as a matrix.

Polypeptide masses were analyzed using an AXIMA Resonance MALDI-quadrupole ion trap TOF MS (Shimadzu, Japan). The acquisition mass range was 0.75–3.5 kDa in a mid mass positive ion mode. The raw spectrum data were analyzed by a Mascot Distiller peak processing software (Version 2.4.3.3, Matrix Science, UK). Amino acid sequences of the fragmented polypeptides were determined using mostly a peptide mass fingerprinting method (PMF) and if necessary a MS/MS analysis. The sequences were alined against the sequence data in the NCBInr (September 7, 2013; 65,225 non-redundant *At* sequence entries) database on Mascot Server version 2.4.

## Results

### Effect of truncation of A’α-helix

**Fig 2:**
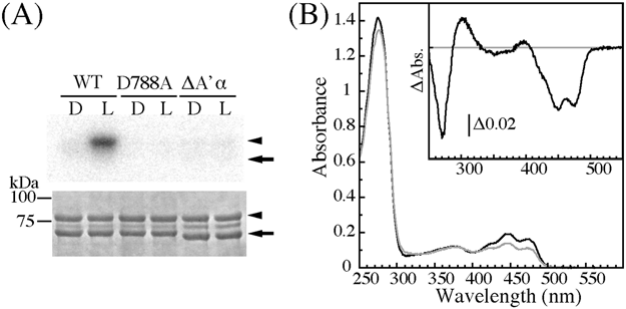
(A) Kinase activity of WT, D788A, and ΔA’α of *At* phot1 LOV2-STK on P1Nt in the dark (D) or under BL irradiation (L). The upper and lower panels indicate autoradiogram and CBB staining of SDS-PAGE gels, respectively. The arrow and the arrowhead indicate the position of LOV2-STK and P1Nt, respectively. (B) Absorption spectra and light minus dark absorption difference spectrum (inset) of ΔA’α in a solution containing 20 mM Tris-HCl, pH 7.8, 500 mM NaCl, 10% (w/v) glycerol, and 1 mM Na_2_EGTA at 20°C. The black and the gray line were measured after dark adaptation and BL irradiation, respectively.

To elucidate the function of A’α-helix, the effect of A’α-helix truncation on the kinase activity was studied (Fig 2A). ΔA’α prepared using the *E*. *coli* expression system was not stable and easily formed aggregates in a 100 mM NaCl buffer solution; therefore, the NaCl concentration was increased to 500 mM to protect against aggregation. The kinase activities of WT, its kinase-dead D788A substitute and ΔA’α were measured using P1Nt as an artificial substrate. In the dark, WT exhibited a faint phosphorylation band compared to D788A whose kinase activity was undetectable. ΔA’α had a similar phosphorylation level to WT in the dark. BL strongly activated the kinase of WT that was impaired by the amino acid substitution at Asp788 to Ala in accordance with previous results [20,38]. In contrast, activation did not occur in ΔA’α indicating that A’α including Glu474 is an essential element for activation. We have previously reported that the kinase activity of *At* phot1 LOV2-STK correlates with the lifetime of S390 in LOV2 and that shortening of the S390 lifetime reduced the kinase activity [20]. Therefore, the kinetics of the photoreaction in ΔA’α was studied (Fig 2B). The ground state absorption spectra of WT and ΔA’α had a similar ratio of the height at 450 nm to 280 nm indicating that they bound a similar amount of FMN. ΔA’α exhibited a characteristic absorption spectral change with a reversible formation of S390 (Fig 2B inset). Its half decay time was calculated as 89 s at 20°C (Table 1). The decay time was almost the same as that of WT in the buffer containing 500 mM NaCl (91 s at 20°C), and the absorption peaks did not shift, indicating that the presence of the A’α-helix does not affect LOV photochemistry. Taken together, these results indicate that the A’α-helix is essential for the intramolecular signaling from LOV2 to the kinase, while it is not involved in the photochemical properties of LOV2 in the LOV2-STK of *At* phot1.

**Table 1:** Half decay times of the dark recovery from S390 to D450 at 20°C.

Sample	T_1/2_ of S390 (s)
WT	59
WT*	91
ΔA’α*	89
D465A	46
E471	36
R472	47
E474A	44
K475A	69
N476A	458

*The sample was dissolved in the buffer containing 0.5 M NaCl.

### Effect of amino acid substitutions in A’α-helix and A’α/Aβ gap

**Fig 3:**
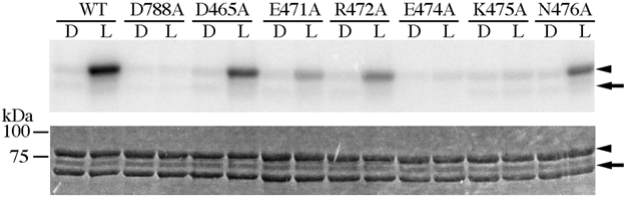
Kinase activity of *At* phot1 LOV2-STK WT and its amino acid substitutes on P1Nt in the dark (D) or under BL irradiation (L). The upper and lower panels indicate autoradiogram and CBB staining of SDS-PAGE gels, respectively. The arrows and the arrow-heads indicate the position of LOV2-STK and P1Nt, respectively.

**Fig 4:**
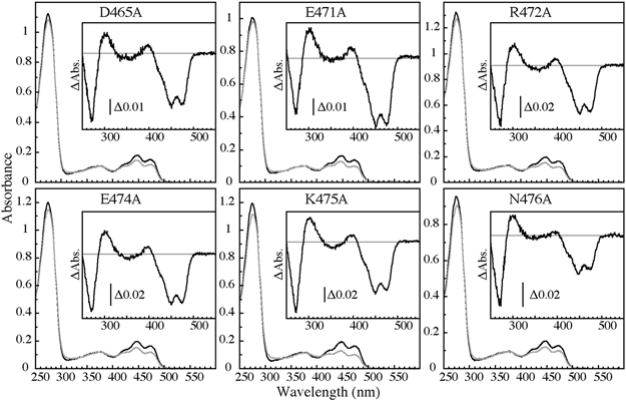
Absorption spectra and of the amino acid substitutes of *At* phot1 LOV2-STK in a solution containing 20 mM Tris-HCl, pH 7.8, 100 mM NaCl, 10% (w/v) glycerol, and 1 mM Na2EGTA at 20°C. The black and the gray line were measured after dark adaptation and BL irradiation, respectively. The insets show the light minus dark absorption difference spectra.

To find key amino acid residues for the signal transduction from LOV2 to STK in the A’α-helix as well as the A’α/Aβ gap, charged amino acids (highlighted in Fig 1) were substituted with alanine and their kinase activities (Fig 3) and photochemistry (Fig 4) were studied. Among the substitutes, D465A in the A’α-helix and N476A in the A’α/Aβ gap preserved similar levels of phosphorylation as WT in a light dependent manner indicating that these amino acid residues do not contribute significantly to the photoactivation of STK. E471A and R472A also exhibited BL-induced phosphorylation; however, their activation levels were lower than of the activation levels of D465A and N476A. A comparison of R472A and E471A demonstrated that R472A has a slightly higher kinase activity than E471A under BL. In contrast, E474A and K475A impaired the BL-induced phosphorylation.

None of the amino acid substitutions prohibited binding FMN to their apoprotein. The absorption spectra of the substitutes in the dark were similar to that of WT indicating that the amounts of bound FMN were comparable among WT and their substitutes (Fig 4). All of the preparations demonstrated a reversible formation of S390 upon BL irradiation that is clearly observed in their light minus dark absorption difference spectra (Fig 4 insets). The half decay times of the substitutes were not so different from that of WT, except for the N476A substitute with a half decay time of 458 s (Table 1).

Substitutions at Glu474 and Lys475 in the A’α/Aβ gap to Ala abolished the BL-induced kinase activation without altering the photochemical properties similarly with the deletion of A’α-helix. This indicates that the pair of amino acid residues are critical for the activation of STK by BL. E471A and R472A substitutions in the A’α-helix resulted in a slight reduction of the BL-induced kinase activation, suggesting minor contributions to STK activation.

### Structural change detected by limited proteolysis

**Fig 5:**
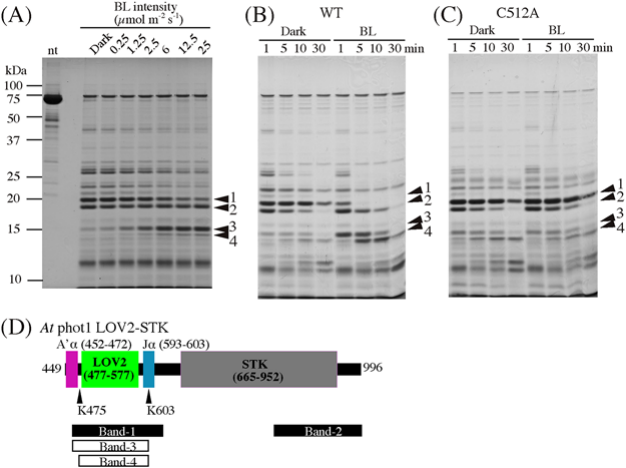
Peptide mapping of *At* phot1 LOV2-STK WT (A) and (B), and C512A (C), by SDS-PAGE after Trypsin digestion in the dark (D) or under BL irradiation (BL). nt indicates the sample without the trypsin-treatment. The gels were stained with CBB. (A) The mapping after BL irradiation with the different fluence rates for 15 min. (B) and (C) show the time courses of the digestion. The four arrowheads indicate the bands of major proteolytic products. (D) Schematic diagram for the positions of the cleavage sites for the four major bands. The black and white bars indicate the proteolytic products obtained in the dark or under BL irradiation, respectively.

To see the BL-induced structural changes in the *At* phot1 LOV2-STK, peptide mapping was performed by limited proteolysis with trypsin. Trypsin digested the WT into two major polypeptides band-1 and band-2 in the dark (Fig 5A). Considering Mass spectrometry and the amino acid sequence of LOV2-STK, we assigned the band-1 and 2 as 463–631 and 835–996, respectively. Band-1 consists of half of the A’α, A’α/Aβ gap, LOV2 and half of the linker between LOV2 and STK including an entire Jα-helix. Band-2 includes the C-terminal half of STK and the C-terminal end part (Fig 5A and 5D, S2 Fig and S2 Table). The two digestion sites, Lys631 in the linker region and Lys835 in the activation loop of STK, do not form a tight structure to protect against trypsin attack in the dark. Under BL, band-1 was degraded further into band-3, 463–603 and band-4, 475–603 (Fig 5A, 5B and 5D). Trypsin digested the substrate in proportion to the BL fluence and the digestion time. To assure the involvement of the photoreaction of LOV2 in these degradations, peptide mapping was performed with a C512A substitute lacking the S390 formation. C512A exhibited a similar SDS-PAGE band pattern to that of WT in the dark, indicating that the Cys512 to Ala substitution does not alter the surface structure of WT in terms of trypsin accessibility (Fig 5). In contrast to WT, C512A did not show marked degradations of band-1 into band-3 and band-4 under BL. Thus, it can be concluded that the formation of S390 causes the conformational changes detectable by trypsin digestion. Because Lys603 is located in the middle of the Jα-helix and Lys475 in the A’α/Aβ gap, these results clearly revealed BL-induced structural changes in the gap as well as in the Jα-helix.

### Structural changes in LOV2-Jα and LOV2-linker

For reference, structural changes in the linker region between the Jα-helix and the STK domain were studied by the peptide mapping of LOV2 with the Jα-helix and LOV2-Jα plus the remaining part of the linker region named LOV2-Jα and LOV2-linker, respectively. Trypsin digested the LOV2-Jα into band-A and B, which were degraded further into band-C and D. BL enhanced the degradation (S3 Fig). Interestingly, the LOV2-linker in the dark showed only the digested band-A, however, further degradation of band-A was not observed. BL induced the degradation of band-A into band-B and C (S3 Fig). This observation suggests a protective role of the remaining linker region against trypsin digestions.

### Effect of amino acid substitution at Glu474 and Lys475 on the structural change

**Fig 6:**
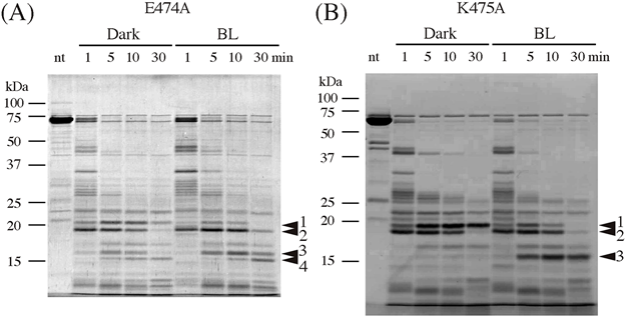
Peptide mapping of *At* phot1 LOV2-STK E474A, (A), and K475A, (B) by SDS-PAGE after trypsin digestion in the dark (D) or under BL irradiation (L). Time courses of the digestion are indicated. nt indicates the sample without the trypsin-treatment. The four arrowheads indicate the bands of major proteolytic products.

To understand the involvement of these structural changes in the kinase activation by BL, peptide mapping was performed with E474A and K475A that impaired light activation of kinase activity. Both substitutes produced bands 1 and 2, which is similar to WT in the dark. This indicates that these amino acid substitutions did not alter the surface structure of WT. BL induced the degradation of band-1 into band-3 and 4 in the E474A, which is similar to WT (Fig 6A). In contrast, K475A exhibited the production of band-3 but not band-4 (Fig 6B). Because K475A lost the trypsin digestion site at Lys475, it is reasonable that band-4 could not be observed. These results demonstrated that BL induces similar structural changes in these substitutes to those of the WT in the A’α/Aβ gap as well as in the Jα-helix; however, it cannot transfer the signal to the later processes responsible for kinase activation by BL.

**Fig 7:**
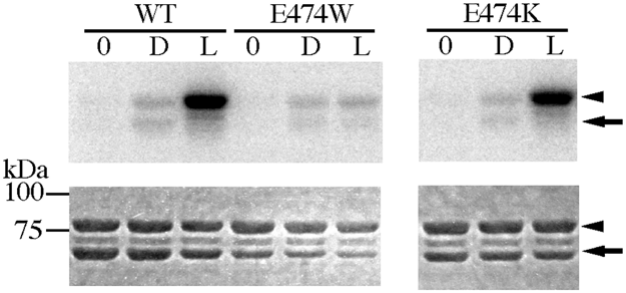
Kinase activity of *At* phot1 LOV2-STK E474W, (A), and K475K, (B) on P1Nt. 0, D and L indicates before incubation, in the dark and under BL irradiation, respectively. The upper and lower panels indicate autoradiogram and CBB staining of SDS-PAGE gels, respectively. The arrow and the arrowhead indicate the position of LOV2-STK and P1Nt, respectively.

The impairment of kinase activation without affecting conformational changes detected by peptide mapping and the photochemical properties implies that Glu474 and Lys475 play special roles in kinase activation. According to the crystal structure of *As* phot1 LOV2-Jα, Lys413 corresponding to the Lys475 in *At* phot1 is supposed to interact with Thr535, which corresponds to Thr604 in *At* phot1, through hydrogen-bonding [28]. In contrast, Glu412 corresponding to Glu474 in *At* phot1 is not involved in hydrogen bonding [28], and Glu474 faces the aqueous phase [36]. This suggests a possible interaction(s) with an unidentified partner(s) in the LOV2-STK that might be involved in signaling kinase activation. To obtain the information regarding this possible interaction, Glu474 was substituted by Trp and Lys. The E474W substitution abolished the BL-induced STK activity but E474K substitution did not (Fig 7). This suggests a neutral hydrophilic amino acid residue is an interacting partner of Glu474 because of the effect of the hydrophobicity and the lack of charge specificity in the amino acid substitution experiments.

## Discussion

### Effect of amino acid substitutions in A’α helix and A’α/Aβ gap on the photochemistry of LOV2

None of the amino acid substitutions nor deletion of the entire A’α helix affected binding of FMN and formation of S390 upon BL excitation (Fig 2B and Fig 4). The decay times of S390 were almost unchanged by the substitutions and the deletion except for the N476A that exhibited approximately an 8 times longer lifetime (Table 1). A similar prolongation was reported with Asn414 to Ala substitute of *As* phot1 LOV2 that corresponds to the *At* phot1 Asn476. The half decay time was prolonged from 80 to 1427 s by the substitution [34,39]. Asn interacts with Gln513 in the Iβ-strand of the *As* phot1 crystal structure [28], which corresponds to the Gln575 in *At* phot1. The Gln is one of the key amino acid residues involved in the photoreaction of LOV2 through the interaction with the C5 oxygen of FMN [22,23,28,39]. Substitution of Gln513 to Leu in *As* phot1 slowed the dark decay by approximately 16 times, although the substitution to Asn did not alter it as much [27]. In contrast, a 16 times acceleration was observed in the Q513D substitute [39]. It has been proposed that the stability of the adduct state is one of the key factors for the decay rate [31,40,41] in which the Gln plays an important role [27,40]. Amino acid residues near the isoalloxazine ring of FMN may be involved in the regulation of the lifetime of S390 through the interaction with key amino acids such as Gln575 in *At* phot. The present results revealed that the amino acids in the A’α-helix and the A’α/Aβ gap do not participate in the regulation of dark decay, except for the Asn476 that potentially interacts with Gln575.

### BL-induced conformational change in Jα-helix

Peptide mapping by limited trypsin digestion clearly showed that BL induced conformational changes in both the Jα-helix and the A’α/Aβ gap in the *At* phot1 LOV2-STK (Fig 5). Trypsin cleaved off the two polypeptides (band-1, 463–631, and band-2, 835–996) of WT (449–996) in the dark. The former revealed that Lys462 in the middle of the A’α-helix and Lys631 in the linker region between the Jα-helix and STK are exposed to aqueous environments in the dark although the Lys462 is located at the middle of the A’α-helix. The dimeric crystal structure of *At* phot1 LOV2-Jα demonstrated that the entire A’α-helix region folds into an α-helix to form the dimeric interface. However, our LOV2-STK preparation exists in a monomeric form in solution [38]. It has been shown that LOV1 of *At* phot1 is in a dimer both in a crystal [42] and in a solution [43]. In contrast, LOV2 of *At* phot1 is in an equilibrium between monomer and dimer in solutions depending on the concentration [24,43]. Digestion at Lys462 suggests that the A’α-helix in our monomeric LOV2-STK in solution may be partially disordered at around the Lys462 in the dark.

Under BL, trypsin digested band-1 firstly at Lys603. Crystal structures of the LOV2-Jα of *At* phot1 [36] indicate that the Lys603 is located at the middle of the Jα-helix. In the *As* phot1 LOV2-Jα with the short A’α-helix, chymotrypsin and trypsin digested at Met530 and Arg521, respectively, in the dark, which was enhanced by BL [25]. Similar enhancement by BL was observed with *At* phot1 LOV2-Jα (S3 Fig). Met530 is located in the middle of the hydrophilic side of the amphiphilic Jα-helix [28]. Digestion at Met530 is in agreement with the present cleavage at Lys603 in the LOV2-STK because both digestions suggest structural changes in the middle of the Jα-helix. However, trypsin did not digest the Lys534 of *As* phot1 that corresponds to Lys603 of *At* phot1, instead, trypsin digested at Arg521 residing next to the N-terminus of the Jα-helix. This may occur because of the different amino acid sequences and constructs of the polypeptides between the *As* LOV2-Jα and the *At* LOV2-STK. A time-resolved NMR study with the *As* phot1 LOV2-Jα polypeptide showed conformational changes and an unfolding of the Jα-helix by BL [31]. The observed BL-induced digestion at Lys603 in *At* phot1 may be derived from these changes in the Jα-helix.

### BL-induced conformational change in A’α/Aβ gap

**Fig 8:**
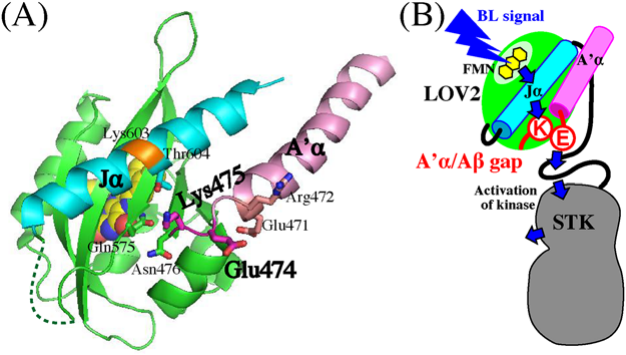
(A) 3D structure of *At* phot1 LOV2-Jα (pdb ID: 4HHD). Key side chains of amino acid residues for intramolecular signaling are indicated with a stick model. FMN is indicated with a space-filling model. A’α-helix and Jα-helix are colored pink and cyan, respectively. The trypsin-digested site at Lys603 is colored orange. (B) Schematic illustration for the hypothetic intramolecular interactions involved in the BL signaling from FMN to STK of *At* phot1. Blue arrows indicate the intramolecular signaling. For details, see the “Discussion”.

After digestion in the Jα-helix under BL irradiation, trypsin digested at Lys475 in the A’α/Aβ gap that connects the A’α-helix and LOV2 and is located 9 Å apart from Lys603 [38] (Fig 8A). In the *As* phot1 LOV2-Jα, chymotrypsin and trypsin digested at Leu408 and Arg410 corresponding to Leu470 and Arg472 in *At* phot1 in the dark were also enhanced under light irradiation suggesting conformational changes in the C-terminal region of the A’α-helix (Fig 1). The lengths of the A’α-helix differ between the *At* and the *As* polypeptides; however, these results suggest that the protein structure is changed by BL in the region from the C-terminal region of the A’α-helix to the A’α/Aβ gap.

A comparison of the order of the digestions in the Jα-helix and the A’α/Aβ gap suggests that the conformational changes in the Jα-helix precedes those in the A’α/Aβ gap. A TG study on the *At* LOV2-Jα, including the full-length A’α-helix, showed that the changes in the Jα-helix occurs faster than those in the A’α-helix based on the comparison between the deleted polypeptides in each helix [37]. This observation is consistent with the present order of the digestion although the Lys475 itself is not included in the A’α-helix. In contrast, MD calculation with the helix-deleted polypeptides based on the crystal structure of *As* phot1 LOV2-Jα with the short A’α-helix suggests that the changes in the A’α-helix precedes those in the Jα-helix [27]. The deletion may result in a different mode of conformational change in the remaining helix because the interactions between the two helices were missing in the calculations. This could explain the discrepancy in the order of the structural changes in the two helices.

BL-induced digestion increased the quantity of band-3 in a fluence-dependent manner (Fig 5) that is similar to the BL fluence-dependent activation of the kinase in the LOV2-STK [20]. This supports the hypothesis that the BL-induced conformational change in Jα-helix is one of the key events for the activation of STK. Based on SAXS analyses, we have demonstrated that *At* phot2 LOV2-STK with lacked kinase activity has a cylindrical shape with a radius of gyration (*Rg*) of 32.4 Å in the dark, whereas the cylinder elongates to show the *Rg* of 34.8 Å under BL [44]. This change might reflect the observed conformational changes in the Jα- and the A’α-helices and the A’α/Aβ gap.

### Essential roles of Glu474 and Lys475 in BL signaling to STK domain

The disappearance of the kinase activation in E474A and K475A (Fig 3) indicates their critical role in the light regulation of kinase activity. However with substitutions, the photoreaction (Fig 4) and BL-induced conformational changes (Fig 6) remained intact indicating that they prohibited the transduction of the observed BL-induced structural changes to the downstream processes that are critical for kinase activation. The process may be mediated firstly by the conformational change at Lys603 in the Jα-helix followed by the change Lys475 in the A’α/Aβ gap (Fig 5). These involvements of Glu474 and Lys475 suggest that the A’α/Aβ gap serves as a joint to connect the intramolecular BL signal from the Jα-helix to the STK domain. The amino acid sequence for the present A’α-helix truncation included the Glu474. The observed impairment of the kinase activation (Fig 2) may be explained by this as well as the destruction of the intact conformation required for the signaling via the gap by truncation.

In the dimeric *At* phot1 LOV2-Jα crystal structure [36], Thr604 in the Jα-helix locates near the side chain of Lys475 in the A’α/Aβ gap in the dark (Fig 8A). The interaction between Thr and Lys is observable in the monomeric LOV2-Jα of *As* phot1 in the dark [28]. BL displaced the N- and C-terminal flanking regions of LOV2 through rearrangement of the hydrogen-bonding network [28]. This rearrangement may be related to the observed structural change (Fig 5) in the Jα-helix and the A’α/Aβ gap. In contrast, Glu474 turns its side chain toward the external aqueous phase [28] (Fig 8A) suggesting a possible interaction between Glu474 and a hypothetical partner amino acid residue in the other part, but not in the A’α, LOV2 and Jα-helix. The protection effect of the linker against trypsin attack (S3 Fig) suggests that the hypothetical partner, possibly a neutral hydrophilic amino acid residue, might exist in the linker region that may interact with the Glu474 through a hydrogen bond-like interaction (Fig 7). The SAXS model for the *At* phot2 LOV2-STK proposes the location of the linker between LOV2 and STK domain [44] that may explain this protection effect (Fig 8B). Glu471 and Arg472 in the C-terminal part of the A’α-helix slightly reduced kinase activation (Fig 3). Glu471 interacts with the side chains of Asp494 in the Cα-helix and Arg504 in the Dα-helix, while Arg472 forms the dimer interface [36] that is not the case with the monomeric LOV2-STK [36]. Arg472 may face to the solution phase (Fig 8A). The reduction of kinase activation suggests that the C-terminal region of the A’α-helix also has a minor contribution in connecting the BL signal from the Jα-helix to the STK domain.

## Conclusion

A hypothetical intramolecular signaling pathway from the FMN to the STK in *At* phot1 is proposed based on the present study, and the known information is illustrated schematically in Fig 8B. The isoalloxazine ring of the FMN bound in the α/β pocket of the LOV2 forms hydrogen bond networks among the near amino acid residues in the dark [22,23,28,36]. BL induces a flipping of Gln575 residue on the Iβ strand of the β-sheet that initiates a structural change cascade in LOV2 through rearrangement of the hydrogen bond network [26]. The structural change in the β-sheet [28] brings the dissociation and the unfolding of the Jα-helix that is anchored mainly by the hydrophobic interactions between the Jα-helix and the β-sheet in which Ile608 play an important role in the dark [29–31]. This structural change propagates to Glu474 and Lys475 in the A’α/Aβ gap that interacts with the Jα-helix and possibly with the amino acid(s) in the linker region in the dark. The BL signal alters the interactions of the A’α/Aβ gap with the linker that is essential for the activation of the kinase, in which the C-terminal part of the A’α-helix including Glu471 and Arg472 exhibits a minor contribution. So far, many studies have proposed the important roles of the Jα and A’α helices during the BL-signaling processes. In addition, the present study clearly demonstrated the essential role of the A’α/Aβ gap including Glu474 and Lys475 as a joint in the BL signaling from the Jα-helix and the A’α-helix to STK in signal transduction. The BL signal may propagate downstream from the Jα-helix with the aid of the A’α/Aβ gap that activates STK.

Two amino acid residues, Glu474 and Lys475, in the gap are conserved among higher plants; however, they are not conserved in the bacterial LOV proteins that do not have an STK signaling module. Crystal structures of bacterial LOV proteins, such as the LOV-STAS protein of *E*. *coli* YtvA [45], the EL222LOV-HTH protein of a marine bacterium *Erythrobacter litoralis* HTCC2594 [46], the PpSB1-LOV protein of a gram-negative bacterium *Pseudomonas putida* KT2440 [47] and the LOV-HK protein of a pathogenic bacteria *Brucella abortus* [48], do not demonstrate the same interactions between the Jα-helix and A’α-helix that is observed in the crystal structures of the higher plant phots [28,36] although some of them have both the A’α and the Jα-helices [46,47]. Accordingly, the function of the joint seems to be a characteristic of higher plant phots that have an STK signal-transmitting module. Our kinase assay system using the LOV2-STK enables us to obtain further insight into the molecular processes underlying signal transduction to the STK domain. The information concerning these signaling processes will provide useful information for designing a new light-regulatable molecular switch [49].

## Supporting Information

S1 Fig Estimation of molecular weight of mutants by size exclusion column chromatography. Ovalbumin (44 kDa), Conalbumin (75 kDa), Aldolase (158 kDa) and Ferritin (440 kDa) were used for the molecular weight standards (open black circle). The other symbols, see the Figure.
(TIF)

S2 Fig Mass spectra of trypsin-digested polypeptides from band-1 (A) and band-2 (B) in Fig 5 (A). (TIF)

S3 Fig Time course of the peptide mapping of *At* phot1 LOV2-Jα, (A), and LOV2-linker, (B). Samples were digested by trypsin in the dark (D) or under BL irradiation (L). The four arrowheads indicate the bands of major proteolytic products.
(TIF)

S1 Table Primer sets used for site-directed mutagenesis. (DOCX)

S2 Table Assignment of polypeptides of band-1 and band-2 in S1 Fig. (DOCX)
