# Supplementary figures and images for: Essential Role of the A’α/Aβ Gap in the N-Terminal Upstream of LOV2 for the Blue Light Signaling from LOV2 to Kinase in Arabidopsis Photototropin1, a Plant Blue Light Receptor
Source: PLoS One. 2015 Apr 17;10(4):e0124284. doi: 10.1371/journal.pone.0124284 (PMC4401697; doi:10.1371/journal.pone.0124284)

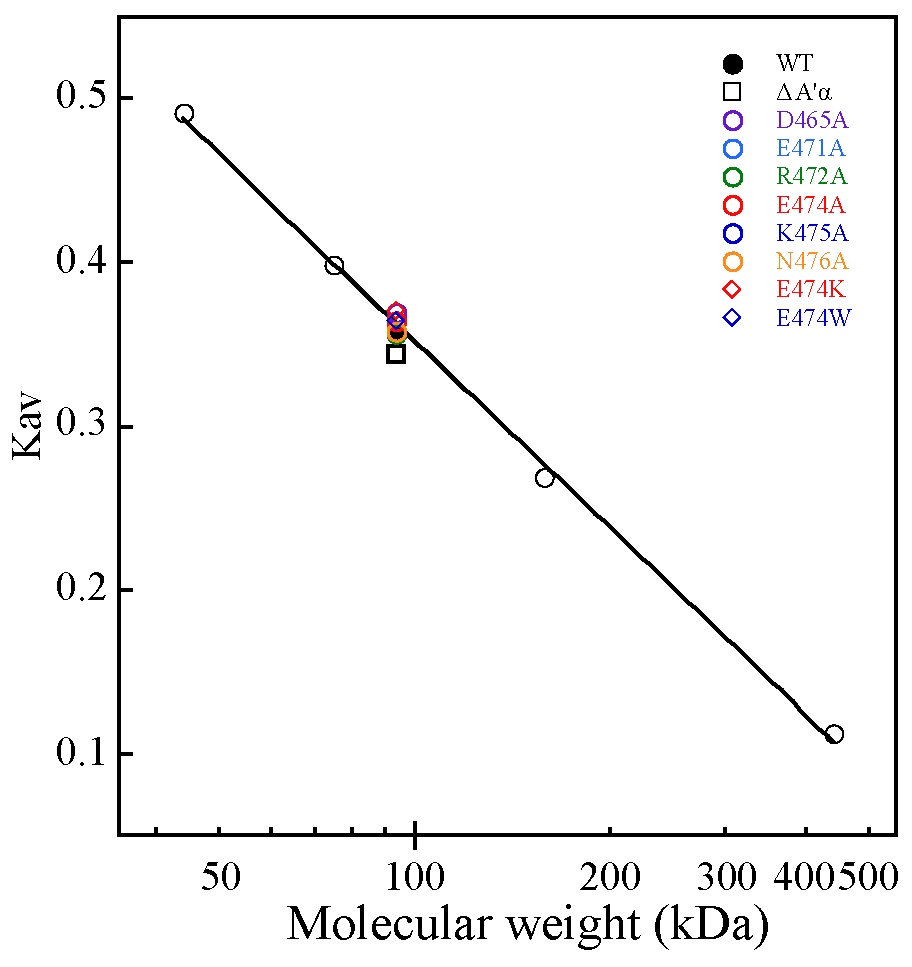

Supplement: S1 Fig — Ovalbumin (44 kDa), Conalbumin (75 kDa), Aldolase (158 kDa) and Ferritin (440 kDa) were used for the molecular weight standards (open black circle). The other symbols, see the Figure. (TIF) [file pone.0124284.s001.tif]

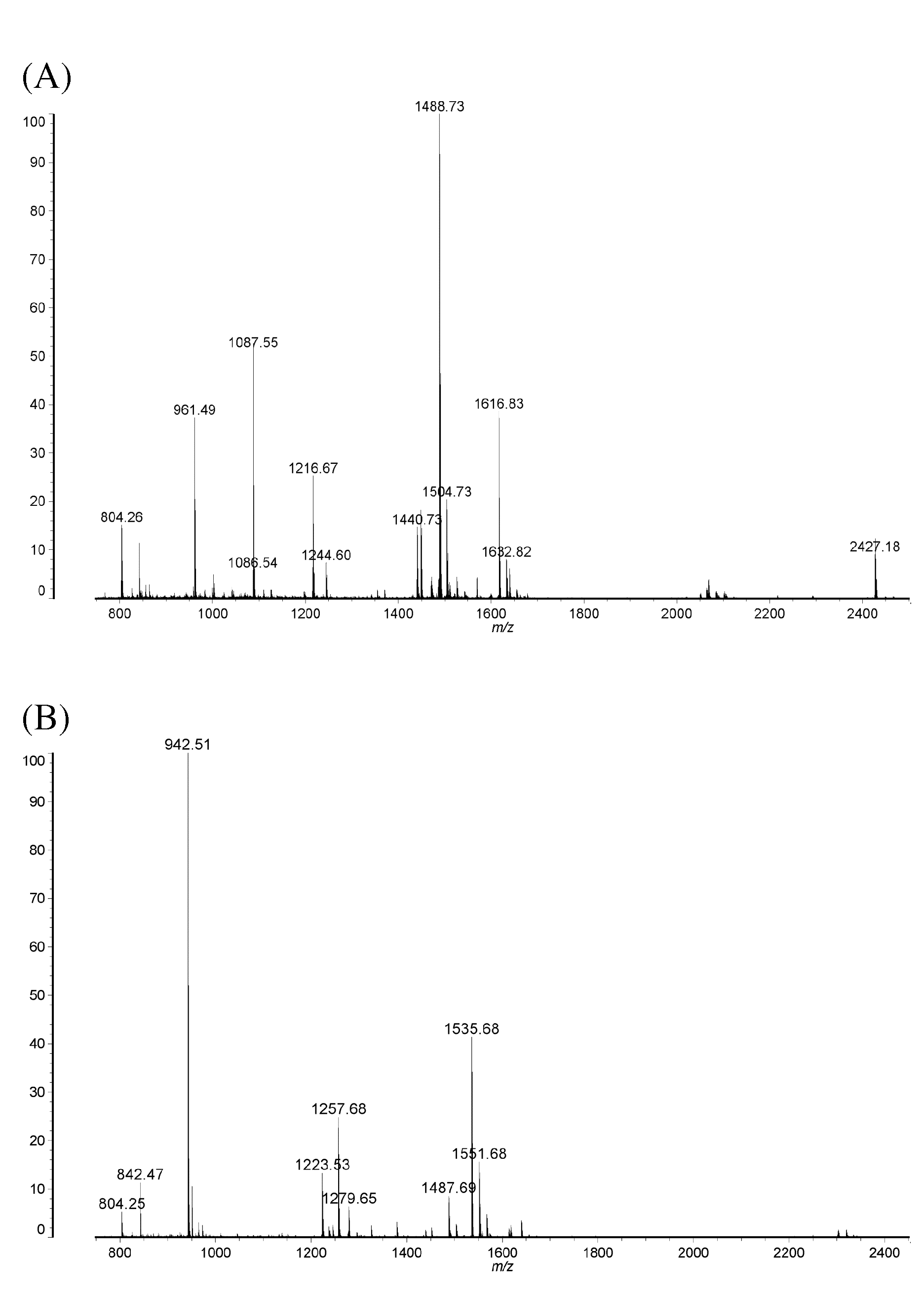

Supplement: S2 Fig — (TIF) [file pone.0124284.s002.tif]

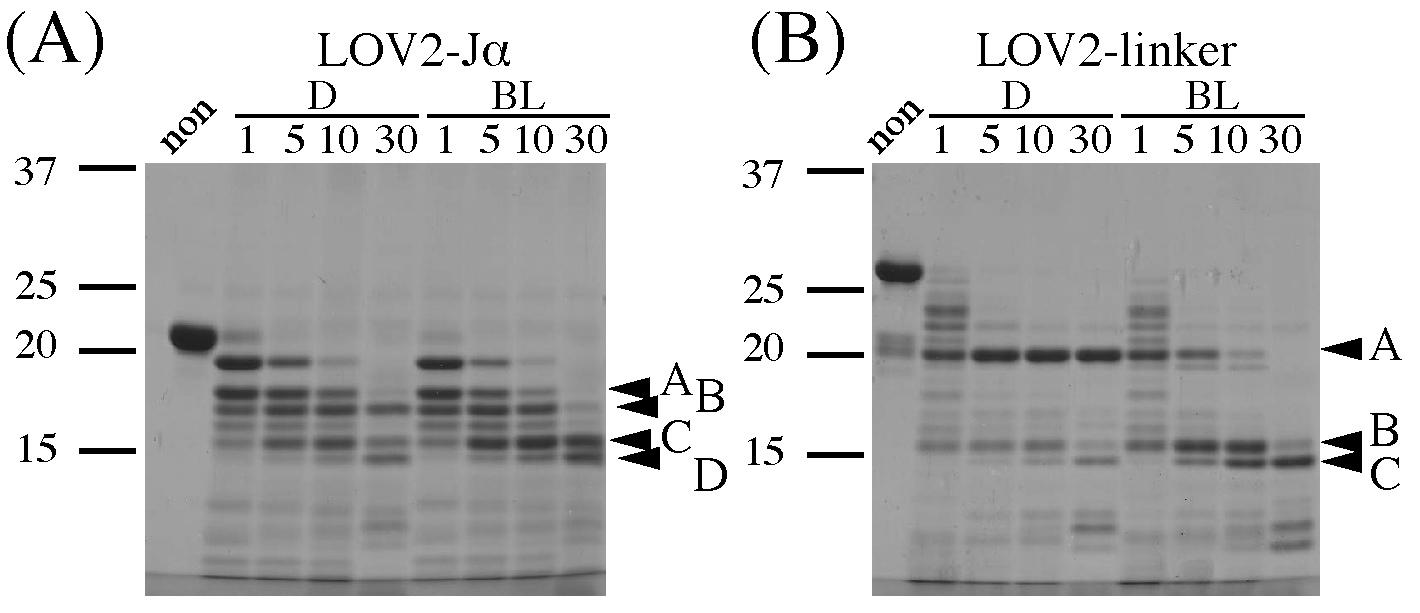

Supplement: S3 Fig — Samples were digested by trypsin in the dark (D) or under BL irradiation (L). The four arrowheads indicate the bands of major proteolytic products. (TIF) [file pone.0124284.s003.tif]
