# Supplementary material for: Essential Role of the A’α/Aβ Gap in the N-Terminal Upstream of LOV2 for the Blue Light Signaling from LOV2 to Kinase in Arabidopsis Photototropin1, a Plant Blue Light Receptor
Source: PLoS One. 2015 Apr 17;10(4):e0124284. doi: 10.1371/journal.pone.0124284 (PMC4401697; doi:10.1371/journal.pone.0124284)
Supplement: S1 Table — (DOCX) [file pone.0124284.s004.docx]

S1. Table. Primer sets used for site-directed mutagenesis.

| Mutation | Primer |
| --- | --- |
| ΔA’α | 5’-GCTACTACACTCGAACGTCATATGAAGAATTTCGTCATC-3’ |
|  | 5’-GATGACGAAATTCTTCATATGACGTTCGAGTGTAGTAGC-3’ |
| D465A | 5’-GAGAAAGGGTATTGCTCTAGCTACTACACT-3’ |
|  | 5’-AGTGTAGTAGCTAGAGCAATACCCTTTCTC-3’ |
| E471A | 5’-AGCTACTACACTCGCACGTATCGAGAAGAA-3’ |
|  | 5’-TTCTTCTCGATACGTGCGAGTGTAGTAGCT-3’ |
| R472A | 5’-ACTCGAACGTATCGCGAAGAATTTCGTCAT-3’ |
|  | 5’-ATGACGAAATTCTTCGCGATACGTTCGAGT-3’ |
| E474K | 5’-ACACTCGAACGTATCAAGAAGAATTTCGTC-3’ |
|  | 5’-GACGAAATTCTTCTTGATACGTTCGAGTGT-3’ |
| E474W | 5’-ACACTCGAACGTATCTGGAAGAATTTCGTCAT-3’ |
|  | 5’-ATGACGAAATTCTTCCAGATACGTTCGAGTGT-3’ |
| K475A | 5'-GAACGTATCGAGGCTAATTTCGTCATC-3' |
|  | 5'-GATGACGAAATTAGCCTCGATACGTTC-3' |
| N476A | 5'-CGTATCGAGAAGGCTTTCGTCATCACT-3' |
|  | 5'-AGTGATGACGAAAGCCTTCTCGATACG-3’ |
