# Supplementary material for: Essential Role of the A’α/Aβ Gap in the N-Terminal Upstream of LOV2 for the Blue Light Signaling from LOV2 to Kinase in Arabidopsis Photototropin1, a Plant Blue Light Receptor
Source: PLoS One. 2015 Apr 17;10(4):e0124284. doi: 10.1371/journal.pone.0124284 (PMC4401697; doi:10.1371/journal.pone.0124284)
Supplement: S2 Table — (DOCX) [file pone.0124284.s005.docx]

S2 Table. List of MS-sequenced polypeptide band-1 (A) and band-2 (B) in Figure 5 (A).

| (A) Polypeptide band-1 | | | | |
| --- | --- | --- | --- | --- |
| *M*_r_ observed | Polypeptide sequence | Start/End  residue# | *M*_r_ calculated | Delta |
| 1216.67 | KGIDLATTLER | 462/472 | 1215.68 | -0.02 |
| 1088.58 | GIDLATTLER | 463/472 | 1087.59 | -0.01 |
| 961.49 | NFVITDPR | 476/483 | 960.50 | -0.02 |
| 2427.18 | LPDNPIIFASDSFLELTEYSR | 484/504 | 2426.20 | -0.02 |
| 1448.74 | FLQGPETDLTTVK | 514/526 | 1447.76 | -0.02 |
| 2064.02 | NAIDNQTEVTVQLINYTK | 530/547 | 2063.05 | -0.04 |
| 1616.83 | KFWNIFHLQPMR | 551/562 | 1615.84 | -0.02 |
| 1488.73 | FWNIFHLQPMR | 552/562 | 1487.75 | -0.02 |
| 1639.81 | GEVQYFIGVQLDGSK | 566/580 | 1638.83 | -0.03 |
| 1002.53 | NVIEETAVK | 587/595 | 1001.54 | -0.02 |
| 1087.56 | TAVNIDEAVR | 604/613 | 1086.57 | -0.02 |
| 2067.92 | ELPDANMTPEDLWANHSK | 614/631 | 2066.94 | -0.03 |

| (B) Polypeptide band-2 | | | | |
| --- | --- | --- | --- | --- |
| *M*_r_ observed | Polypeptide sequence | Start/End  residue# | *M*_r_ calculated | Delta |
| 1535.68 | SQQTPIFMAEPMR | 835/848 | 1534.73 | -0.05 |
| 950.49 | TFTNVLQK | 900/907 | 949.52 | -0.04 |
| 1613.89 | DLKFPASIPASLQVK | 908/922 | 1612.92 | -0.04 |
| 1379.63 | RLGCFEGANEVK | 936/947 | 1378.67 | -0.04 |
| 942.51 | GINWALIR | 954/961 | 941.54 | -0.04 |
| 2318.96 | CTNPPELETPIFSGEAENGEK | 962/982 | 2318.04 | -0.08 |
| 1617.76 | VVDPELEDLQTNVF | 983/996 | 1616.79 | -0.04 |
